# 

**Published:** 1905-06-17

**Authors:** 


### NOTICE TO CORRESPONDENTS.

All MSS., Letters, Books for Review, and other matters intended for the Editor should be addressed The Editor, "The Hospital," The Hospital Buildings, 28 & 29 Southampton Street, Strand, W.C.

The Editor cannot undertake to return rejected MS., even when accompanied by stamped directed envelope.

**Notice to Advertisers and Subscribers.**

All Advertisements, Orders for Copies of The Hospital, and Business Communications should be addressed to **THE MANAGER (Not to the Editor),** The Hospital, 28 & 29 Southampton Street, Strand, London, W.C.

The Cost of Subscription, post free, is at follows :—Per week, 3½d.; per uarter, 3s. 3d.; per half-year, 6s. 6d.; per annum, 13s. Foreign and olonial :—Per annum, 19s. 6d., payable, in all cases, in advance.

**NOTICE.—Vol. XXXVII. of "The Hospital" October 1904 to March 1905), in handsome cloth binding, will shortly be on sale at the office of this Journal, price 10s. 6d. Cases for binding the Numbers contained in this Volume 2s. Index to Vol. XXXVII., 3½d., post free.**

**The Monthly Part for June is now ready. Price 1s., by post, 1s. 3d.**

### MEDICAL APPOINTMENTS.

**W. P. Birmingham**, M.D.Dub., Official Visitor to the Fremantle Lunatic Asylum and Claremont Hospital for the Insane, West Australia. **H. Cardin**, M.R.C.S., L.R.C.P.Lond., Certifying Surgeon under the Factory and Workshop Act for the Stock District of the county of Essex. **A. E. Clark**, M.B., Ch.B.Glasg., reappointed Surgeon to the Home and Dispensary of the Royal Infirmary, Glasgow. **Ernest Francis Clowes**, L.R.C.P.Lond., M.R.C.S., Medical Officer for the Charfield and Tortworth Districts by the Thornbury (Gloucestershire) Board of Guardians for three years. **G. H. Davies**, L.R.C.P.&S.Edin., Honorary Consulting Ophthalmic Surgeon to the Blackburn and East Lancashire Infirmary. **A. Dewar**, M.D., C.M.McGill Univ., L.S.A.Lond., Medical Superintendent of the Royal Hamadryad Seamen's Hospital, Cardiff. **A. Duncan**, M.D.Lond., Physician to the Western General Dispensary. **W. McAdam Eccles**, M.S.Lond., F.R.C.S., Examiner in Surgery to the Society of Apothecaries. **E. D. Scott Heyliger**, B.A., M.B., C.M.Glasg., Honorary Ophthalmic Surgeon to the Blackburn and East Lancashire Infirmary. **William R. Jackson**, M.D., C.M.Edin., District Medical Officer to the Liskeard (Cornwall) Board of Guardians. **T. Henry Jones**, M.D.Edin., D.P.H.Cantab., Education Medical Officer for Surrey. **D. N. Knox**, M.B., C.M.Glasg., reappointed Surgeon to the Home and Dispensary of the Royal Infirmary, Glasgow. **D. C. McVail**, M.B.Glasg., reappointed Physician to the Home and Dispensary of the Royal Infirmary, Glasgow. **Walter Edward Manby**, B.A., M.B., B.Ch.Cantab., L.R.C.P.Lond., M.R.C.S., District Medical Officer and Medical Officer to the Workhouse by the Bridport Board of Guardians. **Patrick Joseph Moloney**, L.K.Q.C.P.Irel., District Medical Officer, Wyndham; Quarantine Officer for the Port of Wyndham, and Public Vaccinator for the Urban, Suburban, and Rural Districts of Wyndham, West Australia. **T. M. Ness**, M.D.Edin., Honorary Physician to Margaret Street Hospital for Consumption and Diseases of the Chest, Margaret Street, Cavendish Square, W. **Herbert Robert Cambridge Newman**, M.B.Durh., L.R.C.P.Lond., M.R.C.S., Medical Officer to the Bristol Dispensary. **W. O'Connor**, L.R.C.P., L.R.C.S.Irel., Certifying Surgeon under the Factory and Workshop Act for the New Ross District of the county of Wexford. **W. J. O'Sullivan**, L.R.C.P.&S.Edin., L.F.P.S.Glasg., Certifying Surgeon under the Factory and Workshop Act for the Lisdoonvarna District of the county of Clare. **D. Wells Patterson**, M.B., Durh., Honorary Assistant to the Department for Diseases of the Skin in the Royal Infirmary, Newcastle-on-Tyne. **Alan H. Pinder**, M.R.C.S., L.R.C.P., Assistant House Surgeon to the Royal Southern Hospital, Liverpool. **Philip Prebble**, M.B., M.Ch.Aberd., Honorary Assistant Physician to the Blackburn and East Lancashire Infirmary. **A. Gardner Robb**, M.B.R.U.I., D.P.H., Visiting Medical Superintendent of the City Fever Hospital, Belfast. **Nigel F. Stallard**, M.B.Lond., Honorary Visiting Physician to Fairlight Hall Convalescent Home, Hastings (in connection with Margaret Street Hospital for Consumption and Diseases of the Chest, London, W.). **A. Wallace Taylor**, L.D.S., R.C.S.Eng., Honorary Dental Surgeon to the Children's Convalescent Home at Weston-super-Mare, [in connection with the Bristol Royal Hospital for Children and Women. **Daniel Gilbert Miller Teague**, M.B., Ch.M.Edin., Officer of Health, Wiluna, West Australia. **George Henry Temple**, M.B., C.M.Edin., Honorary Medical Officer to the Western-super-Mare Crèche. **R. F. Tobin**, F.R.C.S.Irel., Consulting Surgeon to the City Hospital for Diseases of the Skin, and Honorary Surgeon to St. Vincent's Hospital, Dublin. **George Henry Trenfield**, L.R.C.P.Lond., M.R.C.S., Medical Officer for the Eighth District by the Bristol Board of Guardians. **A. M. Ware**, M.A., M.D., B.Sc.Camb., Honorary Physician to Margaret Street Hospital for Consumption and Diseases of the Chest, Margaret Street, Cavendish Square, W. **A. L. Watson**, M.B., Ch.B.Glasg., Anæsthetist to the Home and Dispensary of the Royal Infirmary, Glasgow. **Eric M. Wilkins**, M.B., Ch.B., Accident House Surgeon at the Manchester Royal Infirmary.

### A Dozen Special Text-Books for Nurses.

| | | |
|---|---|---|
| 1. | HOW TO BECOME A NURSE. The Nursing Profession: How and Where to Train. New Edition. Edited by Sir HENRY BURDETT, K.C.B. | 2/- net, 2/4 post free. |
| 2. | HOW TO BECOME A CERTIFIED MIDWIFE. By E. L. C. APPEL, M.B. | 1/- net, 1/1 post free. |
| 3. | A COMPLETE HANDBOOK OF MIDWIFERY. By Dr. J. K. WATSON. Specially designed to meet the requirements of the Central Midwives Board. Illustrated with 143 Diagrams, &c. | 6/- net, 6/4 post free. |
| 4. | HOSPITAL SISTERS AND THEIR DUTIES. By EVA C. E. LÜCKES. Third Edition. | 2/6 post free. |
| 5. | OPHTHALMIC NURSING. By SYDNEY STEPHENSON, M.B. New and Revised Edition. Illustrated. | 3/6 net, 3/10 post free. |
| 6. | NURSING IN DISEASES OF THE THROAT, NOSE, & EAR. By P. MACLEOD YEARSLEY. Illustrated. | 2/6 post free. |
| 7. | ART OF MASSAGE. By Mrs. CREIGHTON HALE. Second Edition. Illustrated. | 6/- post free. |
| 8. | ART OF FEEDING THE INVALID. New and Popular Edition. Bound in cloth. | 1/6 post free. |
| 9. | PRACTICAL GUIDE TO SURGICAL BANDAGING AND DRESSINGS. By WM. JOHNSON SMITH, F.R.C.S. Suitable size for the apron pocket. Profusely Illustrated. | 2/- post free. |
| 10. | THE EXAMINATION OF THE URINE. By J. K. WATSON, M.D. | 1/- net, 1/1 post free. |
| 11. | THE MODERN NURSING OF CONSUMPTION. By Dr. JANE H. WALKER. | 1/- net, 1/2 post free. |
| 12. | ON PREPARATION FOR OPERATION IN PRIVATE HOUSES. By a SURGEON. | 6d. post free. |

**The Scientific Press, Ltd.,** Publishers of Nursing Text-Books, Case-Books & Charts of all descriptions,

28 & 29 SOUTHAMPTON STREET, STRAND, LONDON, W.C.

### The NURSING MIRROR.

**The Nurse's Own Paper.**

**Price ONE PENNY Weekly.**

Of all Booksellers and Newsagents, or posted direct from the Office.

*Write for Subscription Rates to The Manager,*

THE NURSING MIRROR, The Hospital Building, 28 & 29 Southampton Street, Strand, London, W.C.

